# Identification of critical genes and molecular pathways in COVID-19 myocarditis and constructing gene regulatory networks by bioinformatic analysis

**DOI:** 10.1371/journal.pone.0269386

**Published:** 2022-06-24

**Authors:** Fengjun Zhang, Cheng Yu, Wenchang Xu, Xiao Li, Junchen Feng, Hongshuo Shi, Jingrong Yang, Qinhua Sun, Xianyi Cao, Lin Zhang, Min Peng

**Affiliations:** 1 College of Acupuncture and Massage, Shandong University of Traditional Chinese Medicine, Jinan, China; 2 Department of Traditional Chinese Medicine, Shandong Provincial Hospital affiliated to Shandong First Medical University, Jinan, China; 3 Department of Traditional Chinese Medicine, Shandong University of Traditional Chinese Medicine Affiliated Hospital, Jinan, 250014, Shandong, China; 4 Department of Cardiology, Shandong University of Traditional Chinese Medicine Affiliated Hospital, Jinan, 250014, Shandong, China; 5 College of Traditional Chinese Medicine, Shandong University of Traditional Chinese Medicine, Jinan, China; 6 First Clinical Medical College, Shandong University of Traditional Chinese Medicine, Jinan, China; 7 Department of Clinical Pharmacy, Shaoxing People’s Hospital, Shaoxing Hospital, Zhejiang University School of Medicine, Shaoxing, China; University of Hail, SAUDI ARABIA

## Abstract

**Background:**

There is growing evidence of a strong relationship between COVID-19 and myocarditis. However, there are few bioinformatics-based analyses of critical genes and the mechanisms related to COVID-19 Myocarditis. This study aimed to identify critical genes related to COVID-19 Myocarditis by bioinformatic methods, explore the biological mechanisms and gene regulatory networks, and probe related drugs.

**Methods:**

The gene expression data of GSE150392 and GSE167028 were obtained from the Gene Expression Omnibus (GEO), including cardiomyocytes derived from human induced pluripotent stem cells infected with SARS-CoV-2 in vitro and GSE150392 from patients with myocarditis infected with SARS-CoV-2 and the GSE167028 gene expression dataset. Differentially expressed genes (DEGs) (adjusted P-Value <0.01 and |Log2 Fold Change| ≥2) in GSE150392 were assessed by NetworkAnalyst 3.0. Meanwhile, significant modular genes in GSE167028 were identified by weighted gene correlation network analysis (WGCNA) and overlapped with DEGs to obtain common genes. Functional enrichment analyses were performed by using the "clusterProfiler" package in the R software, and protein-protein interaction (PPI) networks were constructed on the STRING website (https://cn.string-db.org/). Critical genes were identified by the CytoHubba plugin of Cytoscape by 5 algorithms. Transcription factor-gene (TF-gene) and Transcription factor-microRibonucleic acid (TF-miRNA) coregulatory networks construction were performed by NetworkAnalyst 3.0 and displayed in Cytoscape. Finally, Drug Signatures Database (DSigDB) was used to probe drugs associated with COVID-19 Myocarditis.

**Results:**

Totally 850 DEGs (including 449 up-regulated and 401 down-regulated genes) and 159 significant genes in turquoise modules were identified from GSE150392 and GSE167028, respectively. Functional enrichment analysis indicated that common genes were mainly enriched in biological processes such as cell cycle and ubiquitin-protein hydrolysis. 6 genes (*CDK1*, *KIF20A*, *PBK*, *KIF2C*, *CDC20*, *UBE2C*) were identified as critical genes. TF-gene interactions and TF-miRNA coregulatory network were constructed successfully. A total of 10 drugs, (such as Etoposide, Methotrexate, Troglitazone, etc) were considered as target drugs for COVID-19 Myocarditis.

**Conclusions:**

Through bioinformatics method analysis, this study provides a new perspective to explore the pathogenesis, gene regulatory networks and provide drug compounds as a reference for COVID-19 Myocarditis. It is worth highlighting that critical genes (*CDK1*, *KIF20A*, *PBK*, *KIF2C*, *CDC20*, UBE2C) may be potential biomarkers and treatment targets of COVID-19 Myocarditis for future study.

## Introduction

Coronavirus disease 2019 (COVID-19) has been defined as a global pandemic by the WHO since March 2020 and is still ravaging the world with high morbidity and mortality. Globally, as of 5:20 pm CET, 18 February 2022, there have been 418,650,474 confirmed cases of COVID-19, including 5,856,224 deaths, reported to WHO (https://covid19.who.int). The common clinical manifestations of SARS-CoV-2 infection are pneumonia, fever, cough, myalgia, and fatigue, and in severe cases, respiratory distress and lymphopenia, with complications including respiratory distress syndrome, secondary infections, and acute heart injury [1].

Numerous studies have shown that [2–4] ACE2 is one of the potential pathogenic targets of SARS-CoV-2 which uses serine protease to activate the S protein to bind to the ACE2 receptor of the cell and enter the cell for virus transmission [5]. ACE2 is broadly expressed in various tissues including the lung, heart, and kidney [6–8], which make these tissues at a higher risk of infection with the new coronavirus. Further studies showed that SARS-CoV-2 binding to receptor proteins in target cells resulted in reduced ACE2 expression levels [9] (low levels of ACE2 are a risk factor for heart disease [6]) and TLR4 activation is a potential mechanism leading to cardiac diseases, especially myocarditis [10]. This is consistent with the prevalence of myocardial injury in COVID-19 patients [11, 12].

With the increase in clinical cases [13, 14] and reported deaths [15] of SARS-CoV-2 associated respiratory and cardiac complications, people increased interest in COVID-19 Myocarditis. However, in clinical practice, the diagnosis of COVID-19 Myocarditis is not well established, and the biological pathways associated with the two are not fully understood, which has caused widespread concern in the medical community [16, 17].

Generally, myocarditis is caused by various viral infections, poisoning, and immune reactions [18]. Although endomyocardial biopsy (EMB) is the gold standard for the diagnosis of myocarditis, it is limited by the level of medical facilities [19] and the requirements of epidemic prevention and control, and there is evidence that EMB has the potential to further aggravate the patient’s condition [17]. On the other hand, a large number of mildly ill patients with clinical symptoms suspicious of COVID-19 Myocarditis are often advised to use non-invasive diagnostic tools such as cardiac magnetic resonance imaging; cardiac magnetic resonance (CMR) imaging is the current non-invasive diagnostic tool for patients with suspected myocarditis [20]. However, for patients with chronic myocarditis, the diagnostic performance of CMR is poor. These add to the difficulty of clarifying the diagnosis of COVID-19 myocarditis [17]. Physicians are often limited to vague diagnoses in clinical decision-making, which may account for the low diagnosis rate of COVID-19 Myocarditis [16]. Consequently, there is a strong need to explore valid, objective, and reliable biomarkers, such as mRNA and protein markers that can be used to diagnose COVID-19 Myocarditis and to explore the pathogenesis of COVID-19 Myocarditis to lay the foundation for further treatment.

Although a large number of antiviral drugs are currently used in the treatment of COVID-19. However, clinical efficacy data for antivirals in patients with COVID-19 Myocarditis are lacking [17]. At present, it is urgent to explore drugs related to the treatment of COVID-19 Myocarditis.

High-throughput screening offers the possibility to screen for mRNA and protein diagnostic markers, clarify biological pathways and relationships in COVID-19 and myocarditis, and screen for gene-targeted drugs.

Our study was conducted by downloading differential analysis of SARS-CoV-2 infected cardiac stem cell data from the GEO database, performing weighted gene correlation network analysis (WGCNA) on human myocarditis dataset to identify disease-related modules and associated genes, and matching with the DEGs to identify the common genes. Kyoto Encyclopedia of Genes and Genomes (KEGG) pathway analysis and Gene Ontology (GO) analysis were performed for the common genes to identify potential biological pathways and pathogenesis. The PPI networks were constructed to screen out critical genes and proteins to clarify disease diagnostic markers to screen out small molecule drugs based on critical genes. Subsequently, based on critical genes, the TF-gene networks and TF-miRNA coregulatory networks were studied for related pathway analysis to lay the foundation for further research and clinical diagnosis and treatment of COVID-19 Myocarditis.

**Fig 1:**
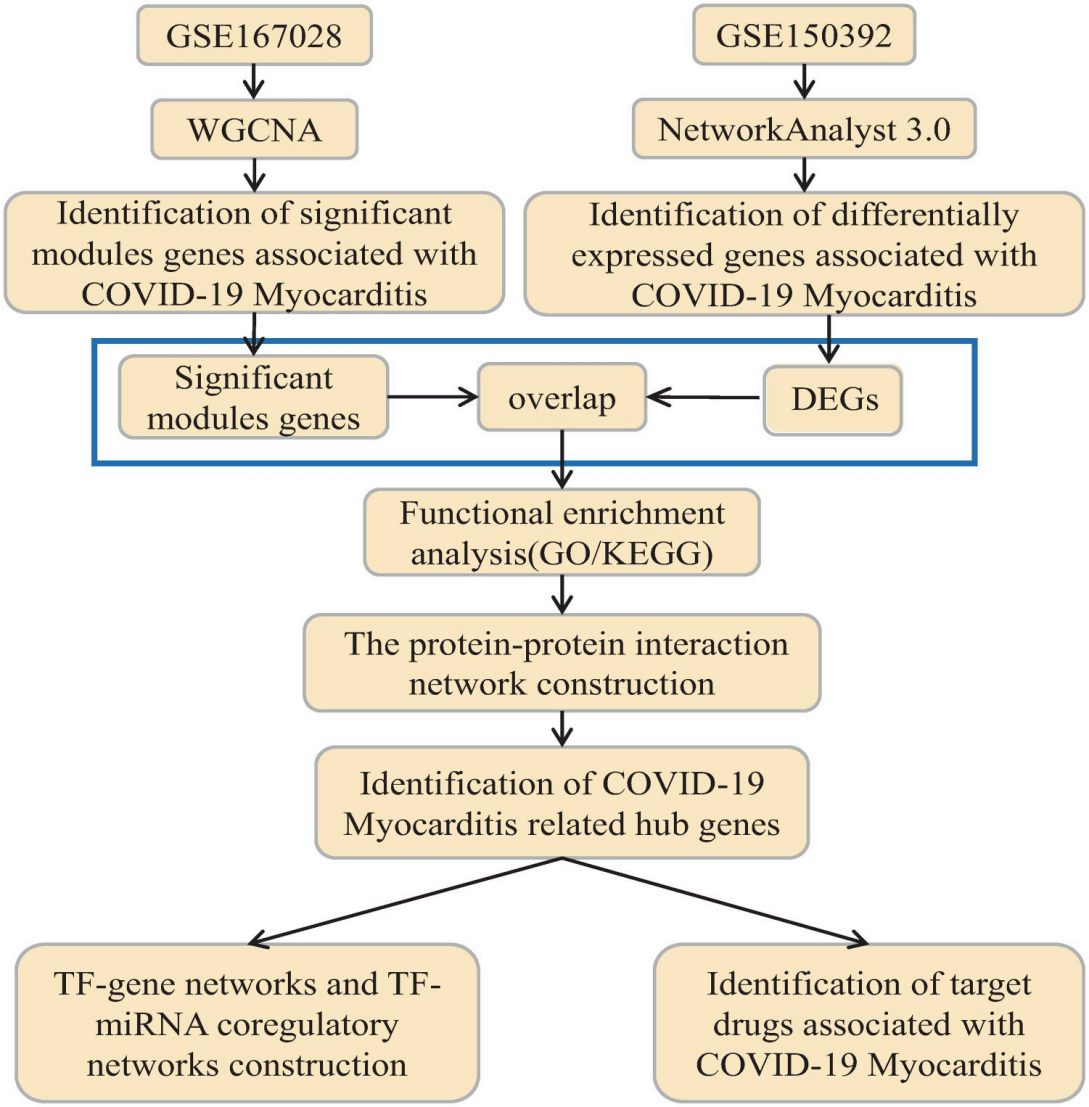
Workflow chart of this study. WGCNA, weighted gene correlation network analysis; DEGs, differentially expressed genes; GO, Gene Ontology; KEGG, Kyoto Encyclopedia of Genes and Genomes; TF, Transcription factor; miRNA, microRibonucleic acid.

The flow chart for this study is presented in Fig 1.

## Materials and methods

### RNA-sequencing data collection

Dataset related to SARS-CoV-2 infected cardiomyocytes was obtained from the Gene Expression Omnibus (GEO) datasets (https://www.ncbi.nlm.nih.gov/gds/) with accession number GSE150392(https://www.ncbi.nlm.nih.gov/geo/query/acc.cgi?acc=GSE150392) [21, 22] which from GPL18573 Illumina NextSeq 500 (Homo sapiens). There were 6 groups of the GSE150392 dataset, including SARS-CoV-2 infected human induced pluripotent stem cell-derived cardiomyocytes (hiPSC-CMs) groups (n = 3) and Mock hiPSC-CMs (n = 3) groups.

### Identification of the DEGs from GSE150392 dataset

NetworkAnalyst 3.0 (https://www.networkanalyst.ca) [23] is a user-friendly online bioinformatics tool for performing comprehensive gene expression analyses, meta-analyses, and network analyses, which agrees with five data inputs, including one or multiple gene lists, single or multiple gene expression data, raw RNA-seq reads, and serial matrix files. This potent online visualization tool integrates transcription factor-gene interaction networks, RNA-gene interaction networks, and other biological regulatory networks which includes data processing, analysis and data update, integrated knowledge base, and synergistic visualization analysis.

GSE150392 dataset was uploaded to the NetworkAnalyst 3.0 for screening and normalizing. The adjusted P-Value (adj P Val) was analyzed to correct for false-positive results in GEO datasets. “adj P Val <0.01 and |Log2 Fold Change|≥2” were set as the threshold values to screen the differential expression of mRNAs. Circular heatmap and gradient volcano plot were generated by using the “gheatmap” function of the R package “ggtree” (version 3.2.1) and “ggplot2” (version 3.3.5), respectively.

### Weighted correlation network analysis (WGCNA) and matching common genes

WGCNA is a systems biology approach to characterize correlation patterns between genes in microarray samples [24]. This analysis method is designed to find co-expressed gene modules, explore the associations between gene networks and phenotypes of interest, and core genes in the network, and this approach is used to identify candidate biomarkers or therapeutic targets.

The normalized gene expression data were downloaded from GSE167028 (https://www.ncbi.nlm.nih.gov/geo/query/acc.cgi?acc=GSE167028) dataset, which included 32 samples and used to construct a co-expression network by using WGCNA (version 1.7.3) package [24] in R-4.1.1. To be clear, WGCNA package doesn’t recommend attempting WGCNA on a data set consisting of fewer than 15 samples. If at all possible, one should have at least 20 samples. We eliminated the KD groups, divided the COVID-19 positive samples into the disease groups, and combined the remaining samples as control groups to maintain study consistency.

According to the correlation of the trait genes, the neighborhood degree of the trait genes was calculated to investigate the co-expression similarity of each module. To identify the association between the general expression module and the clinical group, the p-value, and the correlation coefficient were calculated to visualize the characteristic heatmap of the modules. Modules with a p-value< 0.05 were considered significant.

Finally, a Venn diagram summarizing the overlapping DEGs and significant module genes was generated by using the OmicShare online tool (https://www.omicshare.com).

### Functional enrichment analysis

Gene Ontology (GO) is a widely-used tool to annotate the functions of genes, especially biological pathways (BP), cellular components (CC), and molecular function (MF) [25]. KEGG Enrichment Analysis is a practical resource for analyzing gene function and related high-level genomic function information [26, 27].

GO pathway analysis of common genes was performed using the R package org.Hs.eg.db (version 3.1.0) as background. Meanwhile, the KEGG pathway gene annotations (c2.cp.kegg.v7.4.symbols.gmt subset) were obtained from the Molecular Signatures Database [28] to perform the KEGG pathway analysis. In addition, the gene set enrichment results were obtained by using the R package clusterProfiler (version 3.14.3). GO terms and KEGG pathways with P-value<0.05 were considered as a significant enrichment.

### PPI network construction to identify critical genes and module analysis

The protein-protein interaction (PPI) network was constructed through the STRING (v11.5; https://cn.string-db.org), a web-based tool for detecting protein interactions by uploading the gene dataset [29, 30]. In this study, the interaction score was set at 0.4. Subsequently, the PPI network data was exported into Cytoscape version 3.7.2 for analysis and visualization.

CytoHybba(https://apps.cytoscape.org/apps/cytoHubba/), a plug-in of Cytoscape software, was used to get the top ten genes through 5 different algorithms: Degree, EPC, Closeness, Betweenness, and Stress, separately [31, 32]. The intersecting genes obtained by the above five methods were considered as critical genes and displayed in the form of a Venn diagram generated by the R package “ggVennDiagram” (version 1.2.0) based on the “ggplot2” (version 3.3.5) package of R [33].

### TF-gene networks and TF-miRNA coregulatory networks construction

TF regulates gene expression by binding to specific regions of genes to form feedforward and feedback loops involved in a variety of biological processes and disease processes [34]. On the other hand, TF binds to miRNAs to co-regulate gene expression [35]. In our study, based on critical genes, TF-gene networks and TF-miRNA coregulatory networks were identified by NetworkAnalyst 3.0 (https://www.networkanalyst.ca) [23]. In subsequent work, gene regulatory networks were imported into the Cytoscape 3.7.2 for visualization and analysis.

### Identification of target drugs associated with COVID-19 myocarditis

The Drug Signatures Database (DSigDB) is an online gene set linking drugs and their target genes which contains 22 527 gene sets, consists of 17 389 unique compounds, and covers 19 531 genes [36]. The Enrichr (https://maayanlab.cloud/Enrichr/) website provides links to access to the DSigDB. In this study, critical genes were uploaded to the Enricher website to identify the drugs associated with COVID-19 Myocarditis.

## Results

### Identification of COVID-19 myocarditis related DEGs

The GSE150392 dataset contains 3 SARS-CoV-2 infected pluripotent stem cell-derived cardiomyocytes (hiPSC-CMs) and 3 Mock pluripotent stem cell-derived cardiomyocytes (hiPSC-CMs) for identification of DEGs in COVID-19 Myocarditis. Under the screening criteria, 850 DEGs were obtained, including 449 up-regulated and 401 down-regulated genes (data in S1 Text).

**Fig 2:**
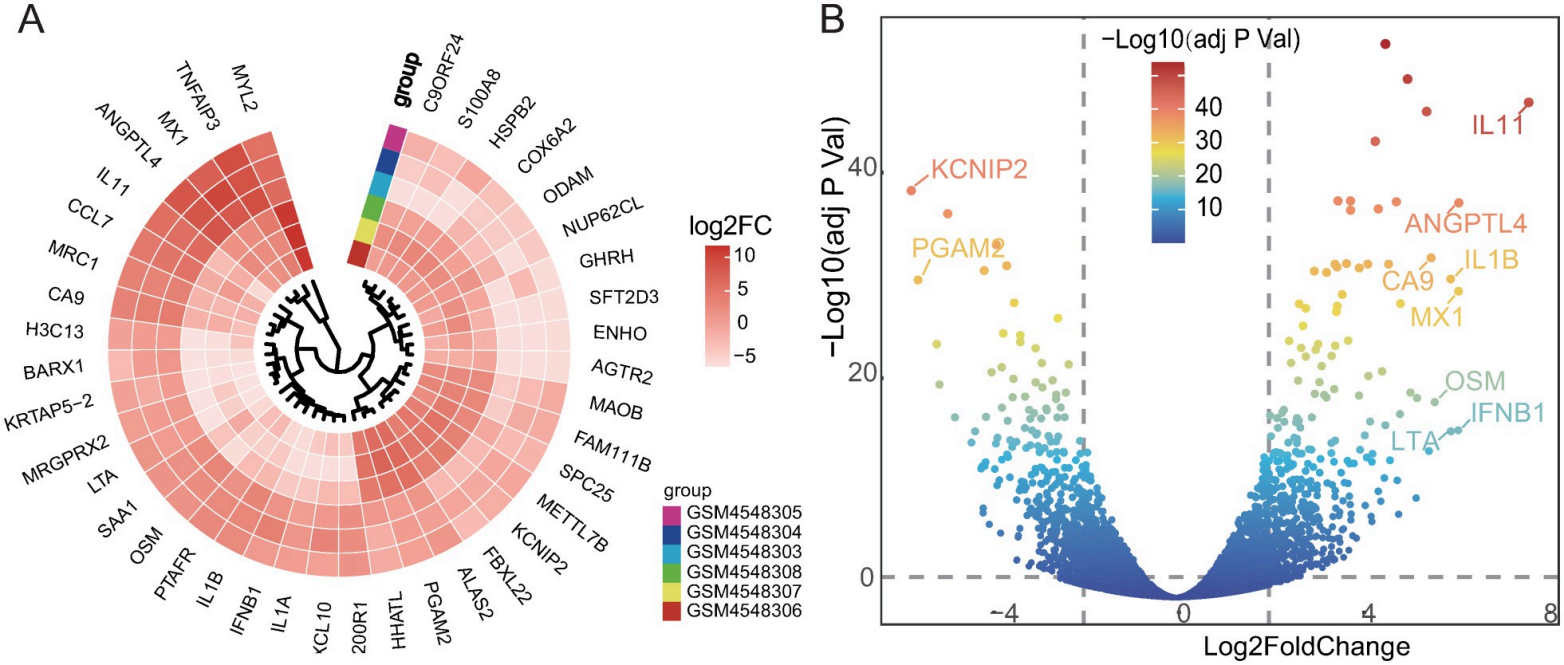
Identification of differentially expressed genes in the GSE150392 dataset by differential analysis. (A) The circular heatmap exhibited the top 40 DEGs sorted by the Log2 FC for the GSE150392 dataset. (B) DEGs in the gradient volcano plot. The top 10 genes were labeled on the plot which was sorted by the Log2 FC and most of them were upregulated (except *KCNIP2* and *PGAM2* which were downregulated). Two vertical lines indicated |Log2 FoldChange| ≥2, severally, and the horizontal line indicated the adj P Val of 0.01. The color of the plots represents the Log2 FoldChange levels [37]. DEGs, differentially expressed genes; FC, fold change; adj P Val, adjusted P-Value.

A gradient volcano plot was exhibited in Fig 2A, which showed the upregulated and downregulated genes that varied as the expression fold changes for the GSE150392 dataset [37]. A circular heatmap exhibited the top 40 DEGs in Fig 2B, while the top 10 DEGs’ details were shown in Table 1.

**Table 1:** The top ten differentially expressed genes (DEGs) in the GSE150392 dataset.

Gene	Log2FoldChange	P-value	Adj P Value	Regulate
*IL11*	7.6191	1.03E-52	5.49E-49	Up
*ANGPTL4*	6.1073	2.08E-42	3.33E-39	Up
*MX1*	6.101	2.39E-33	1.37E-30	Up
*IFNB1*	6.0943	3.20E-19	4.80E-17	Up
*LTA*	5.9337	4.18E-19	6.10E-17	Up
*IL1B*	5.9318	1.43E-34	8.85E-32	Up
*KCNIP2*	-5.7287	8.10E-44	2.17E-40	Down
*OSM*	5.5858	4.27E-22	8.78E-20	Up
*PGAM2*	-5.5819	1.84E-34	1.10E-31	Down
*CA9*	5.5093	7.12E-37	7.62E-34	Up

### Identification of COVID-19 myocarditis associated modules by WGCNA

**Fig 3:**
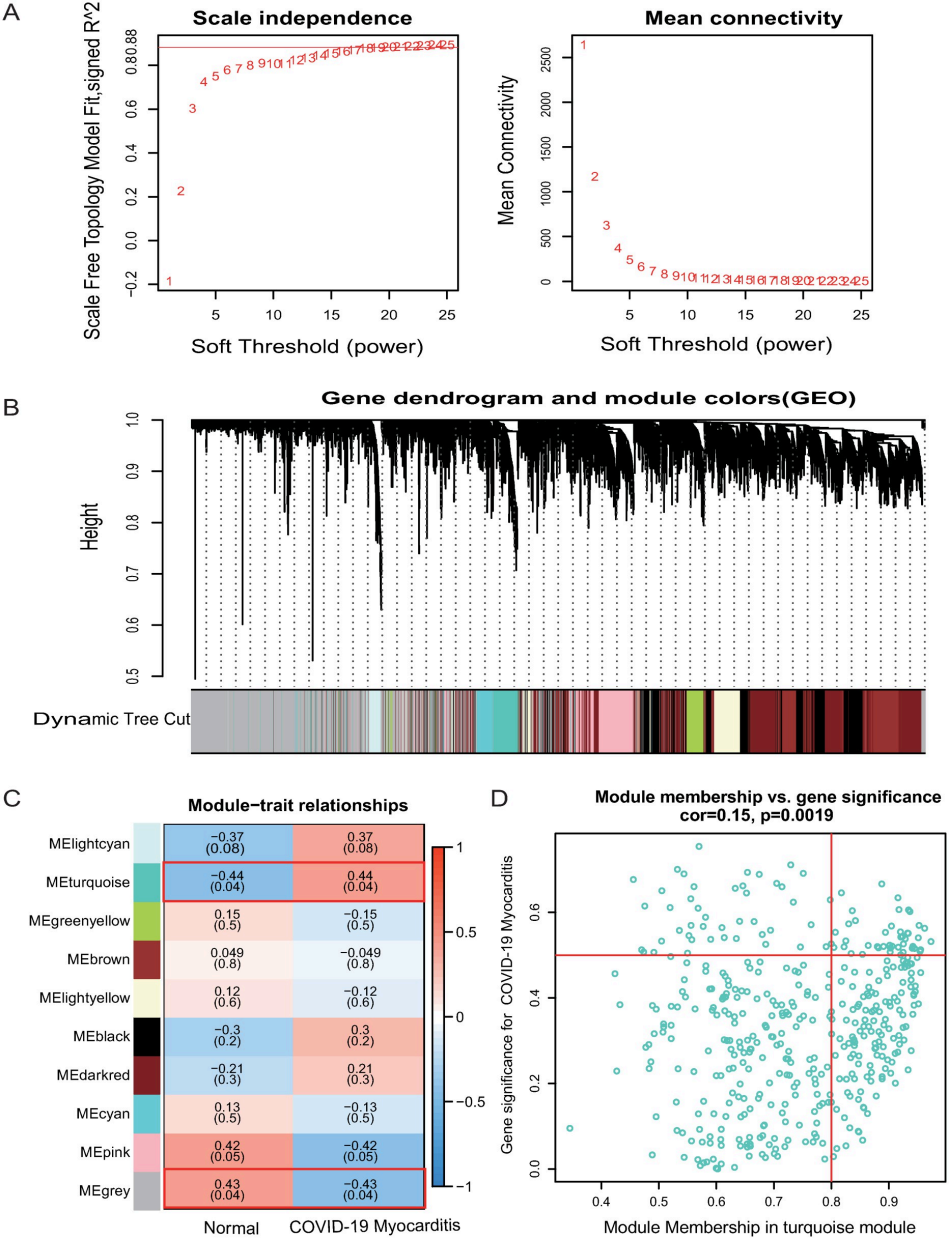
Identification of significant modules and genes of GSE167028 by WGCNA. (A) Network topology analysis with different soft thresholds. The scale-free R^2 was 0.88 and the soft threshold was 20. (B) A cluster dendrogram of module-specific colors showed 10 co-expressed gene modules, each containing more than 80 genes. (C) Correlation between disease groupings and gene modules. (D) The scatter plot of Gene significance vs Module membership in the turquoise co-expression module.

In this study, we constructed a co-expression network for normalized gene expression data of the GSE167028 dataset by the WGCNA package (version 1.7.3) in the R application. The soft threshold was set to 20 to fit a scale-free network and the maximum mean connectivity, while the scale-free R^^2^ was 0.88 (Fig 3A). Meanwhile, a total of 10 co-expression modules, each with more than 80 genes, were identified using the DynamicTreeCut method (Fig 3B). Among the 10 significant modules (Fig 3C), the lightcyan, turquoise (r = 0.15, p = 0.0019, Fig 3D), black, darkred modules were positively related to COVID-19 Myocarditis, whereas the green, brown, lightyellow, cyan, pink, and grey modules were negatively associated with COVID-19 Myocarditis. Nevertheless, only the turquoise module met the criteria which had a p-value of 0.04 (except for the gray module, which contained high amounts of un-classified genes). Therefore, this module was identified as a significant module for further analysis.

We calculated the expression correlation of module feature vectors with genes to obtain module membership (MM) and gene significance (GS). Based on the cut-off criteria |MM| > 0.8 and |GS|>0.2, 159 of 427 genes with high connectivity in the turquoise module were identified.

**Fig 4:**
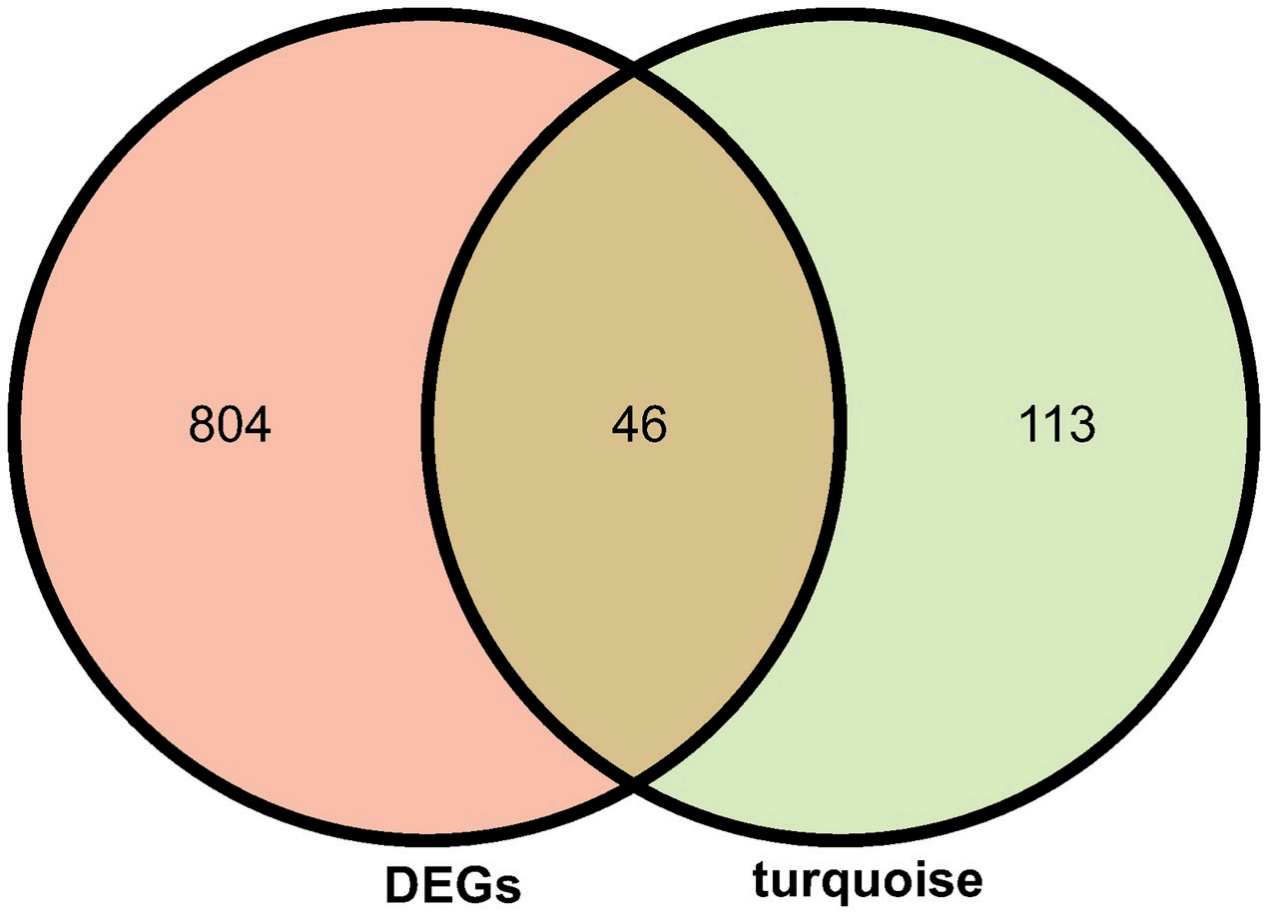
The intersection of DEGs in the GSE150392 dataset and turquoise module genes in the GSE167028. There were 850 DEGs in the GSE150392 dataset and 159 genes in the turquoise module of the GSE167028 dataset, and 150 genes were obtained as common genes by overlapping the two datasets.

Ultimately, 46 common genes were obtained by intersecting with the DEGs of GSE150392 and the genes in the turquoise module of GSE167028 (Fig 4, data in S2 Text).

### Gene function annotations of COVID-19 myocarditis related the common genes

**Fig 5:**
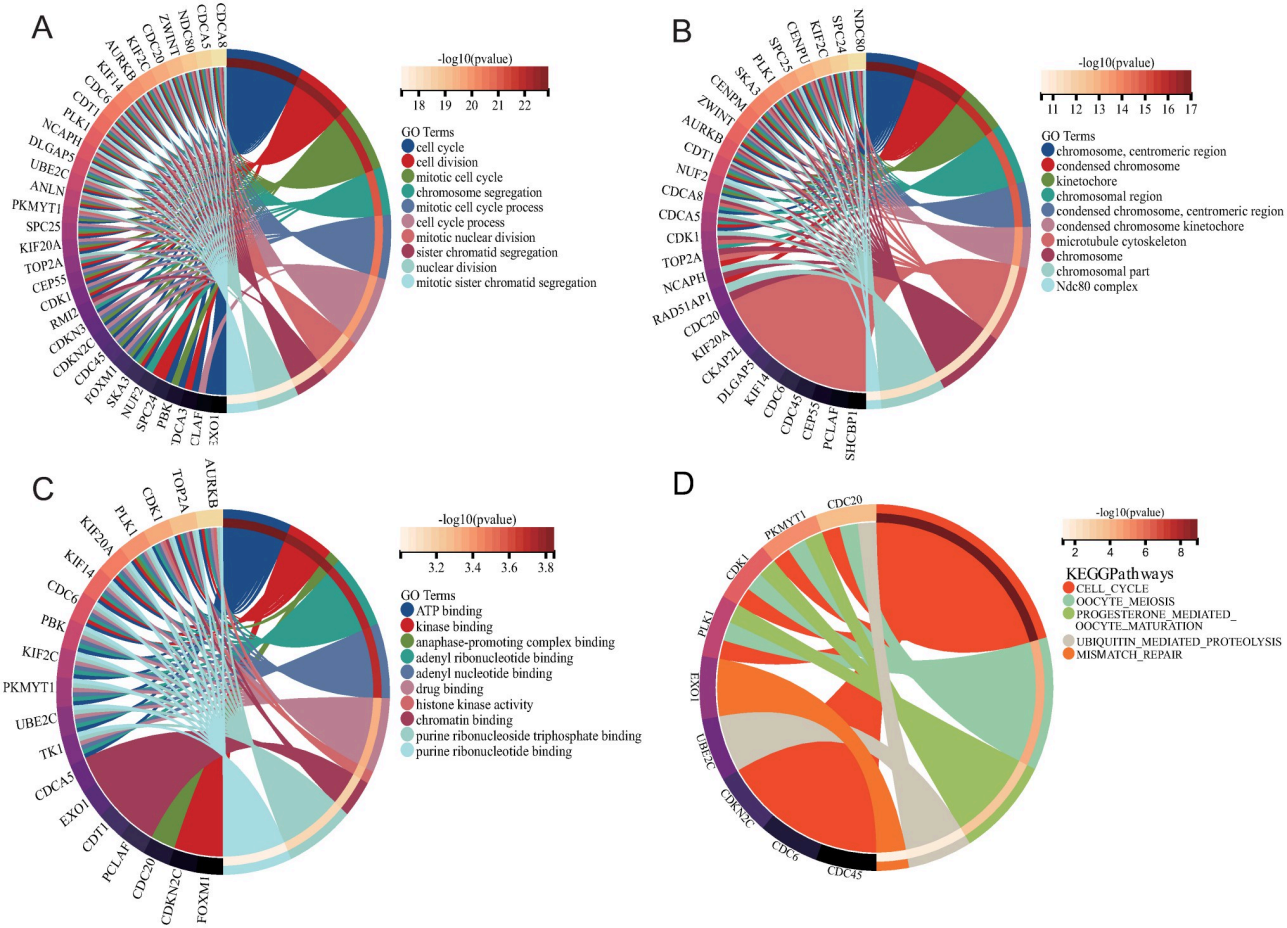
GO and KEGG analysis of COVID-19 myocarditis related to the common genes. (A) Biological Process. (B) Cellular Component. (C) Molecular Function. (D) KEGG pathways analysis.

After obtaining common genes with COVID-19 Myocarditis, GO enrichment and KEGG pathway analysis were performed to understand the biological pathways. The top ten Go terms for biological process, cellular component and molecular function were shown in Table 2 and Fig 5A–5C. The data of GO terms indicated that the common genes were significantly enhanced in cell cycle/division and mitotic cell cycle of biological process. Cellular components revealed significant involvement of microtubule cytoskeleton and chromosome in common genes. For the molecular function subsection, it was apparent that ATP binding was involved in the common genes.

**Table 2:** GO category, GO description, GO ID, and their corresponding P-value.

Category	Description	GO ID	P-value
BP	cell cycle	GO:0007049	1.31E-23
BP	cell division	GO:0051301	3.12E-23
BP	mitotic cell cycle	GO:0000278	1.33E-22
BP	chromosome segregation	GO:0007059	9.27E-22
BP	mitotic cell cycle process	GO:1903047	3.41E-21
BP	cell cycle process	GO:0022402	1.86E-20
BP	mitotic nuclear division	GO:0140014	1.59E-19
BP	sister chromatid segregation	GO:0000819	8.04E-19
BP	nuclear division	GO:0000280	3.57E-18
BP	mitotic sister chromatid segregation	GO:0000070	4.42E-18
CC	chromosome, centromeric region	GO:0000775	9.69E-18
CC	condensed chromosome	GO:0000793	2.73E-17
CC	kinetochore	GO:0000776	2.7E-16
CC	chromosomal region	GO:0098687	1.59E-15
CC	condensed chromosome, centromeric region	GO:0000779	1.99E-15
CC	condensed chromosome kinetochore	GO:0000777	4.25E-14
CC	microtubule cytoskeleton	GO:0015630	3.45E-12
CC	chromosome	GO:0005694	5.64E-12
CC	chromosomal part	GO:0044427	7.3E-12
CC	Ndc80 complex	GO:0031262	3.22E-11
MF	ATP binding	GO:0005524	0.000143
MF	kinase binding	GO:0019900	0.00016
MF	anaphase-promoting complex binding	GO:0010997	0.000166
MF	adenyl ribonucleotide binding	GO:0032559	0.000198
MF	adenyl nucleotide binding	GO:0030554	0.000207
MF	drug binding	GO:0008144	0.000496
MF	histone kinase activity	GO:0035173	0.000534
MF	chromatin binding	GO:0003682	0.000674
MF	purine ribonucleoside triphosphate binding	GO:0035639	0.000737
MF	purine ribonucleotide binding	GO:0032555	0.00099

The next section of the functional enrichment analysis was concerned with the KEGG analysis which was demonstrated in Table 3 and Fig 5D. KEGG analysis found that common genes were mainly enriched in the cell cycle, oocyte meiosis, progesterone mediated oocyte maturation, ubiquitin mediated proteolysis, and mismatch repair.

**Table 3:** KEGG pathways and their corresponding P-values and Q-values, and common genes enriched in their pathways.

KEGG pathways	P-value	Q-value	Gene ID
cell cycle	1.13E-09	5.93E-09	*PLK1*/*CDK1*/*CDC45*/*CDC6*/*PKMYT1*/
			*CDKN2C*/*CDC20*
oocyte meiosis	6.00E-05	1.58E-04	*PLK1*/*CDK1*/*PKMYT1*/*CDC20*
progesterone mediated oocyte maturation	6.17E-04	1.08E-03	*PLK1*/*CDK1*/*PKMYT1*
ubiquitin mediated proteolysis	3.11E-02	4.09E-02	*UBE2C*/*CDC20*
mismatch repair	4.72E-02	4.97E-02	*EXO1*

### Construction of a PPI network and identification of critical genes

**Fig 6:**
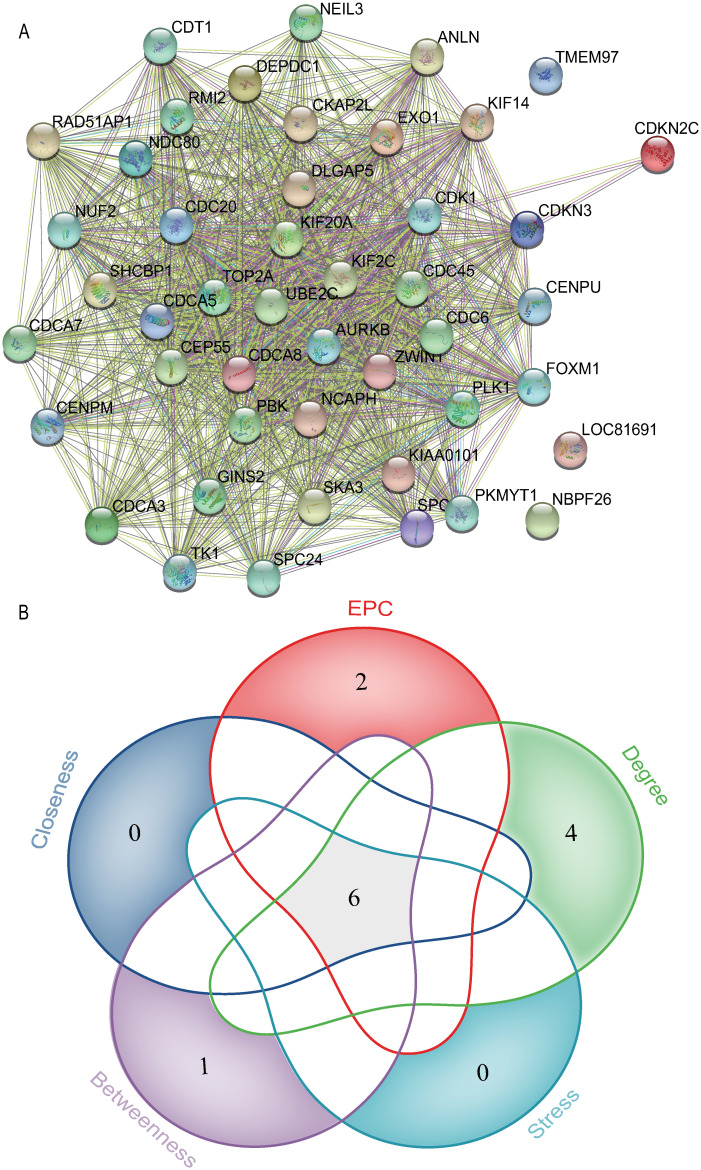
The number of critical genes were shown by using the cytoHubba plugin of cytoscape and the Venn diagram. EPC, Edge Percolated Component.

Among these 46 common genes, a PPI network (46 nodes and 778 edges) was generated by STRRING (v11.5; https://cn.string-db.org). Thereafter, the network was imported into the Cytoscape version 3.7.2 for analysis and visualization. Based on 5 algorithms of the CytoHubba plug-in, six genes (*CDK1*, *KIF20A*, *PBK*, *KIF2C*, *CDC20*, *UBE2C*) were confirmed as critical genes related to COVID-19 Myocarditis (Fig 6). All topological features of critical genes were shown in Table 4. Since these critical genes may be potential biomarkers, they may provide a reference for the diagnosis and treatment of COVID-19 Myocarditis.

**Table 4:** The top 10 genes in the PPI network were calculated using five algorithms.

Gene	Degree	Gene	EPC	Gene	Closeness	Gene	Betweenness	Gene	Stress
***CDK1***	42	***KIF2C***	16.979	***CDK1***	42	***CDK1***	48.66956	***CDK1***	250
***KIF20A***	41	***PBK***	16.82	***KIF2C***	41.5	*CDKN3*	42.99438	*CDKN3*	204
*TOP2A*	41	*RAD51AP1*	16.708	***PBK***	41.5	***KIF2C***	6.66956	***KIF2C***	170
***PBK***	41	***CDK1***	16.659	*RAD51AP1*	41.5	*PBK*	6.66956	***PBK***	170
*AURKB*	41	*CDCA5*	16.594	*CDCA5*	41.5	*RAD51AP1*	6.66956	*RAD51AP1*	170
***KIF2C***	41	*UBE2C*	16.576	***UBE2C***	41.5	*CDCA5*	6.66956	*CDCA5*	170
*CDCA8*	41	*EXO1*	16.562	***KIF20A***	41.5	***UBE2C***	6.66956	***UBE2C***	170
***CDC20***	41	***KIF20A***	16.384	***CDC20***	41.5	***KIF20A***	6.66956	***KIF20A***	170
***UBE2C***	41	***CDC20***	16.344	*DLGAP5*	41.5	***CDC20***	6.66956	***CDC20***	170
*NUF2*	41	*NCAPH*	16.339	*CDC6*	41.5	*DLGAP5*	6.66956	*DLGAP5*	170

### Identification of gene regulatory networks related to critical genes

NetworkAnalyst 3.0 was used to identify the TF-gene networks and TF-miRNA coregulatory networks based on critical genes which were visualized in Fig 8A, 8B.

**Fig 7:**
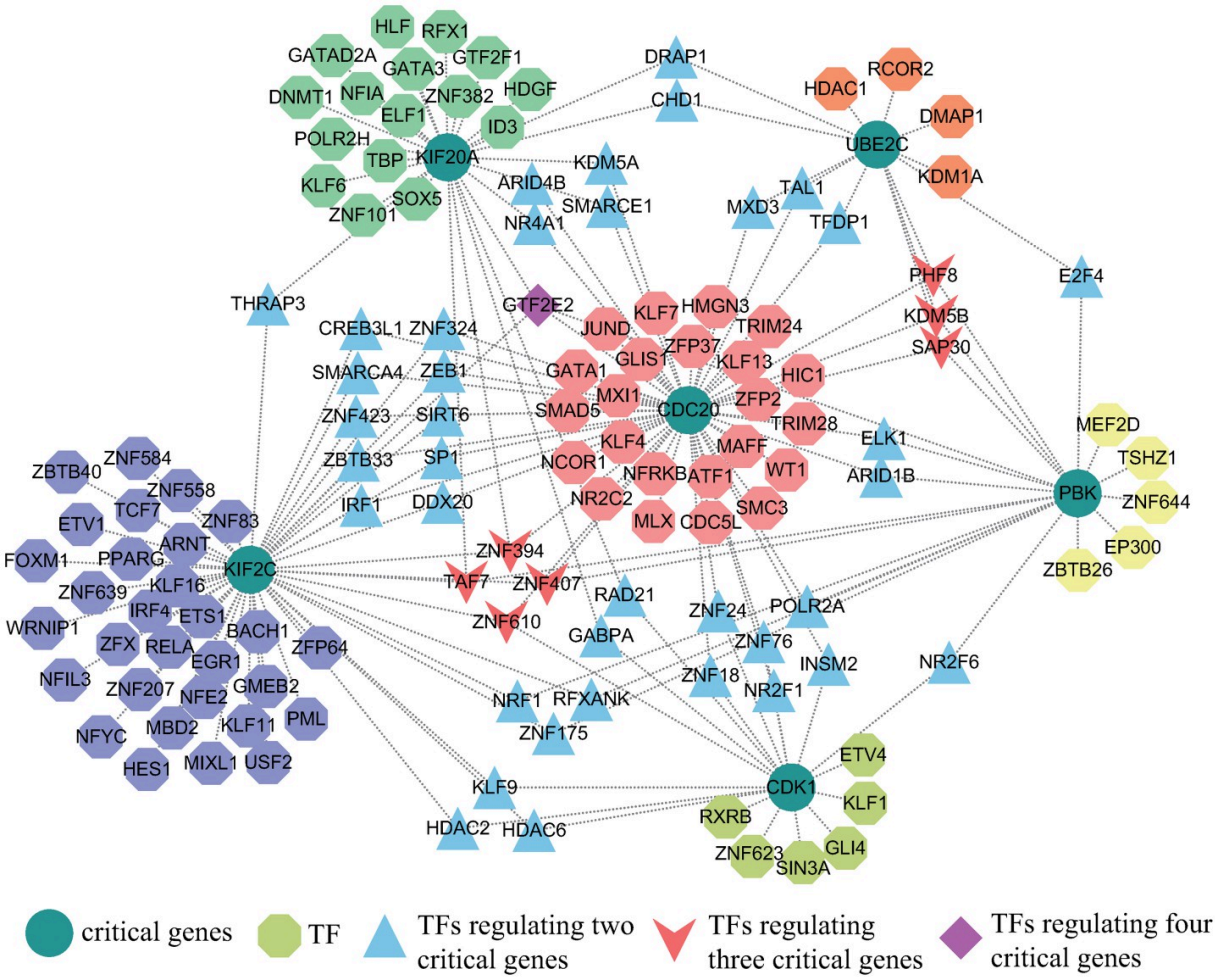
Transcription factor-gene regulatory network in COVID-19 myocarditis. The circular dots represent critical genes, and the octagonal dots attached next to the critical genes represent transcription factors that regulated the critical genes. In addition, the triangular-shaped transcription factors indicated regulation of two critical genes, and the V-shaped transcription factors regulated three key genes. Obviously, the diamond-shaped transcription factor *GF2E2* regulated four critical genes. The network was composed of 142 nodes and 180 edges.

The TF-gene networks comprised 136 nodes and 185 edges. The entire network consists of 130 TF genes and 6 critical genes. *CDC20* was regulated by 55 TF genes and *KIF2C* was regulated by 52 TF genes. In addition, 130 TF genes regulated more than one common gene, which indicated that TF genes were highly regulatory of critical genes. Interestingly, we found that *GTF2E2* had high connectivity in the TF-gene regulatory network, regulating four critical genes simultaneously. Fig 7 showed the TF-gene networks.

**Fig 8:**
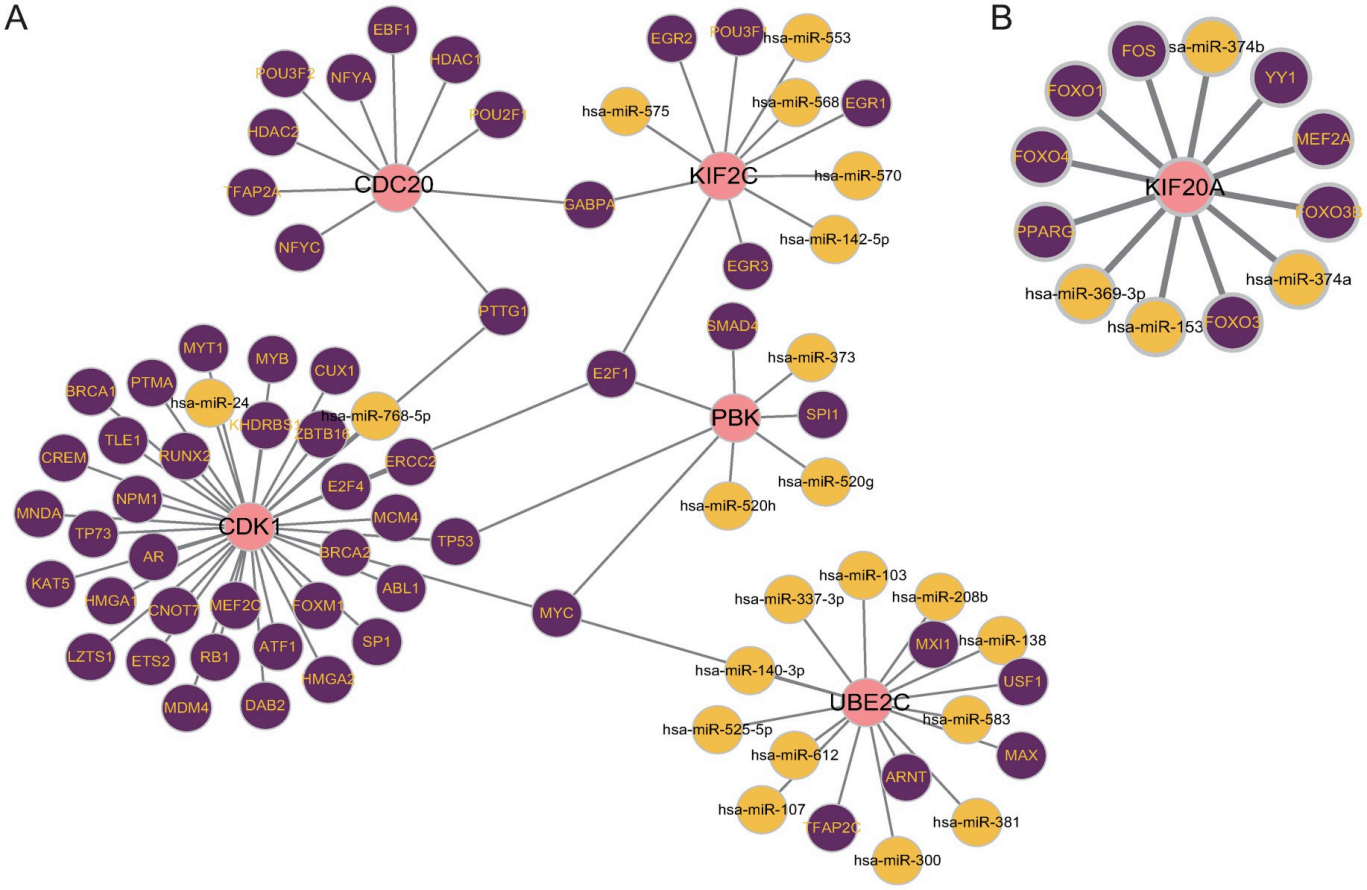
Transcription factors-miRNA-gene regulatory networks in COVID-19 myocarditis. There were two TF-miRNA networks. Pink plots represent critical genes, purple plots represent TF genes, and the others represent miRNAs. Network (A) had 83 plots and 85 edges, which was consisted of 5 critical genes, 56 TF genes, and 21 miRNAs. Network (B) had 13 plots and 12 edges including 1 critical gene, 8 TF genes, and 4 miRNAs.

On the contrary, the TF-miRNA coregulatory networks consist of two parts, one including 83 nodes and 85 edges, and the other including 13 nodes and 12 edges which were shown in Fig 8. A total of 25 miRNAs and 64 TF genes co-regulated critical genes.

### Identification of target drugs associated with COVID-19 myocarditis

Based on critical genes, the drugs related to the COVID-19 Myocarditis were identified by the DSigDB database that was built on the Enrichr website. In the integration of the DSigDB dataset, 308 drug compounds were identified (data in S5 Text). Finally, the top ten drug compounds were screened according to the p-value. Etoposide and methotrexate are two notable genetically linked drug compounds. Meanwhile, *CDC20* and *KIF2C* were associated with the most drug compounds, suggesting that they act prominent roles in drug efficacy. Table 5 showed information on potentially effective drug compounds for COVID-19 Myocarditis.

**Table 5:** COVID-19 myocarditis gene-targeted drugs.

Term	P-value	Combined Score	Genes
etoposide MCF7 DOWN	4.36E-10	19544.37	*CDC20*; *UBE2C*; *KIF2C*; *KIF20A*
methotrexate MCF7 DOWN	6.07E-10	17637.87	*CDC20*; *UBE2C*; *KIF2C*; *KIF20A*
LUCANTHONE CTD 00006227	7.78E-10	9976.195	*CDC20*; *CDK1*; *PBK*; *KIF2C*; *KIF20A*
troglitazone CTD 00002415	1.16E-09	2388335	*CDC20*; *UBE2C*; *CDK1*; *PBK*; *KIF2C*; *KIF20A*
ciclopirox MCF7 DOWN	1.72E-09	12770.53	*CDC20*; *UBE2C*; *KIF2C*; *KIF20A*
5109870 MCF7 DOWN	1.82E-09	12532.66	*CDC20*; *UBE2C*; *KIF2C*; *KIF20A*
thalidomide CTD 00006858	3.51E-08	4975.73	*CDC20*; *UBE2C*; *CDK1*; *KIF2C*
genistein CTD 00007324	5.37E-08	1885048	*CDC20*; *UBE2C*; *CDK1*; *PBK*; *KIF2C*; *KIF20A*
dmnq CTD 00002569	5.41E-08	4339.482	*CDC20*; *UBE2C*; *PBK*; *KIF2C*
testosterone CTD 00006844	5.81E-08	1874705	*CDC20*; *UBE2C*; *CDK1*; *PBK*; *KIF2C*; *KIF20A*

## Discussion

As previously described, a large number of patients with myocarditis were identified during clinical treatment of COVID-19. However, the understanding of COVID-19 and myocarditis is insufficient, and many patients with COVID-19 Myocarditis are not properly diagnosed and well treated. To our knowledge, research on the key genes and pathways by bioinformatics methods between COVID-19 and myocarditis has hardly been reported. The present study was designed to elaborate on the bioinformatics lessons about the key genes and pathways between COVID-19 and myocarditis.

In our study, 850 DEGs and 159 significant module genes from GSE150392 and GSE167028 were identified by bioinformatics-related methods, respectively. For constructing the relationship of the COVID-19 and myocarditis, 46 common genes were overlapped. The remaining studies were functional enrichment analysis, PPI network construction, TF-gene networks, TF-miRNA coregulatory networks construction, and gene targeting drug screening [38]. Eventually, 6 genes (*CDK1*, *KIF20A*, *PBK*, *KIF2C*, *CDC20*, *UBE2C*) were identified by CytoHubba plug-in of Cytoscape as critical genes of COVID-19 Myocarditis for future study.

Based on the common genes, GO terms were identified as a threshold of P-value of < 0.05. According to biological process, the top ten GO terms were cell cycle, cell division, mitotic cell cycle, chromosome segregation, mitotic cell cycle process, cell cycle process, mitotic nuclear division, sister chromatid segregation, nuclear division, and mitotic sister chromatid segregation [39]. The cell cycle is the complete process of cell division and replication and consists of a specific series of events such as cell division, DNA replication, nuclear membrane rupture, spindle formation, and preparation for chromosome segregation [40]. Numerous studies have shown that viruses provide powerful conditions for viral replication and survival by regulating different processes of the cell cycle of host cells [41–44]. For molecular function, ATP binding, kinase binding, anaphase-promoting complex binding, adenyl ribonucleotide binding, adenyl nucleotide binding, drug binding, histone kinase activity, chromatin binding, purine ribonucleoside triphosphate binding, and purine ribonucleotide binding were the top ten GO terms. Adenosine triphosphate (ATP), is an energy metabolite that plays a role in energy transfer and information transmission in various cellular metabolic processes. Recent findings uncovered that SARS-CoV-2 N protein regulates the cell cycle of host cells by specifically binding ATP, which provides us with a new idea to fight against the SARS-CoV-2 pandemic [45]. In addition, the previous study has shown that ATP is also a specific autoantibody for myocarditis [46]. As for cellular components, the top GO terms are chromosome, centromeric region, condensed chromosome, and kinetochore.

The KEGG pathways analysis was achieved from the common genes for identifying similar pathways between COVID-19 and myocarditis. KEGG pathway analysis mainly focused on cell cycle, oocyte meiosis, progesterone mediated oocyte maturation, ubiquitin mediated proteolysis, and mismatch repair, which indicate that they are crucial to the biological progression of COVID-19 Myocarditis. The ubiquitin protein hydrolysis plays an essential role in a range of underlying cellular processes, such as immune responses and inflammatory responses [47]. In addition, it has been shown that ubiquitin protein hydrolysis is associated with myocardial remodelings, such as Atrophy of the heart [48].

PPI network analysis was the most important step in this study, laying the foundation for the subsequent screening of critical genes. Based on topological algorithms (i.e., degree), in this study, *CDK1*, *KIF20A*, *PBK*, *KIF2C*, *CDC20*, and *UBE2C* were identified as critical genes that may be potential biomarkers for COVID-19 Myocarditis.

*CDK1* (Cyclin-dependent kinase 1) plays a critical role in eukaryotic cell cycle control by regulating centrosome cycling and mitotic initiation. There is growing evidence found that *CDK1* can be used as a potential biomarker for a variety of diseases, such as Rhabdomyosarcoma [49], endometrioid endometrial cancer [50]. Furthermore, in recent studies, *CDK1*, promotes the phosphorylation of RAPTOR during mitosis, leading to mTORC1 phosphorylation and affecting the autophagic process [51], which plays an important role in cardiac diseases as a degradation process of cellular self, especially in myocarditis or cardiomyopathy [52]. In addition, *CDK1* regulates the cell cycle leading to cell cycle arrest in cardiomyocytes, the latter being an important factor involved in oxidative stress leading to heart failure [53]. Similarly, in previous studies, *CDK1* was identified as a potential target of COVID-19 [54], so the role of *CDK1* in myocarditis needs to be further investigated.

*KIF20A* (Kinesin Family Member 20A), is a member of the Kinesin-like proteins that play an important role in intracellular transport and cell division [55]. According to the literature, *KIF20A* plays an important role in several cardiovascular diseases, such as restrictive cardiomyopathy and acute type A aortic coarctation. In one case report, exome sequencing analysis of children with congenital cardiomyopathy identified the *KIF20A* complex and in subsequent in vitro experiments in zebrafish, *KIF20A* was identified as the phenotypic gene for cardiomyopathy [56]. Chen et al. found that *KIF20A* was identified as a hub gene involved in the infection of the intestine by the SARS-CoV-2 [57]. All of the above studies provide implications for the study of *KIF20A* in COVID-19 and myocarditis.

*PBK* (PDZ Binding Kinase) plays a regulatory role in cell cycle regulation and cell mitosis. It was reported that using the PathExt tool was able to identify *PBK* as the most common central gene target in activated TopNets to suppress SARS-CoV-2 [58]. Ekaterina et al. showed that genes such as *PBK* regulated myofibril formation and thus caused cardiac hypertrophy [59].

As for the remaining genes, *CDC20* and *KIF2C* were identified as target genes of COVID-19 by bioinformatics means and machine learning [54]. Meanwhile, *UBE2C*, Ubiquitin-Conjugating Enzyme E2 C, is closely related to the cardiovascular system which induced endothelial cell inflammation and endothelial mesenchymal transition, exacerbating aortic sclerosis and calcification [60].

According to critical genes, the TF-gene networks and TF-miRNA co-regulatory networks were established. In our knowledge, transcription factors play important roles in many biological processes by binding specific sequences of genes, such as regulation of gene transcription, control of metabolism, and immune response [61]. And further studies have shown that transcription factors are closely related to a variety of diseases. From the network, it can be seen that *CDC20* has a high rate of interactions with other TF genes. In the TF-gene coregulatory networks, the degree value of *CDC20* was 55. This was closely followed by *KIF2C* with a degree of 52 and *KIF2C* had higher connectivity with *CDC20* with a value of 14. Notably, *GTF2E2* was identified as the transcription factor that regulates the most genes which has been reported to exert inhibitory effects on lung adenocarcinoma in the mTOR pathway. It is well known that the mTOR pathway plays an important role in the autophagic process and the pathogenesis of myocarditis [62], and autophagy has been shown to be closely related to COVID-19 and myocarditis [63], which provides us with a new idea to study the regulatory role of GTF2E2 in COVID-19 Myocarditis. Meanwhile, in the TF-miRNA coregulatory networks, *MYC*, *E2F1*, *PTTG1*, *GABPA*, *TP53* regulated more than one critical gene.

Through the drug database, drug molecules associated with critical genes were identified and sorted by p-value. Etoposide MCF7 DOWN, Methotrexate MCF7 DOWN, Lucanthone CTD 00006227, Troglitazone CTD 00002415, Ciclopirox MCF7 DOWN, STL264925 MCF7 DOWN, Thalidomide CTD 00006858, Genistein CTD 00007324, Dmnq CTD 00002569, Testosterone CTD 00006844 are potentially investigational and therapeutic agents associated with COVID-19 Myocarditis. Because of superior anti-cancer activity, Etoposide plays an important role in cancer treatment. Meanwhile, as a TOP II inhibitor, Etoposide effectively inhibits intracellular replication of SARS-CoV-2’s structural proteins [64] and has a rescue effect on the cytokine storm of the COVID-19 [65]. It has been shown that immunosuppressive therapy has a therapeutic effect on myocarditis and can improve the prognosis of myocarditis [45], which offers the possibility of drug targeting for the treatment of COVID-19 Myocarditis. Methotrexate, an immunosuppressant, also has an inhibitory effect on the COVID-19 cytokine storm [66]. Troglitazone as a type 2 diabetes oral medication, has the effect of improving the sensitivity of muscle and adipose tissue to insulin and inhibiting hepatic gluconeogenesis. A recent study has shown that Troglitazone has the potential to inhibit SARS-CoV-2 NSP9 which plays a vital role in viral replication [67].

Several limitations need to be noted regarding the present study. Firstly, since there are few studies related to COVID-19 Myocarditis, we selected only two datasets from GEO database for bioinformatics study. In addition, due to the limitations of the data set sample, we unified the experimental group in the GSE167028 dataset as the group of patients with myocarditis and unified the healthy adult and pediatric groups as the healthy control group in order to maintain disease grouping consistency and statistical accuracy, which may result in a degree of study heterogeneity. Thirdly, although rigorous bioinformatics analysis was performed in this study, the findings need to be validated with more samples and cellular and animal experiments. A further study with more focus on COVID-19 myocarditis is therefore suggested.

## Conclusion

The bioinformatics study of the GSE167028 and GSE150392 datasets identified 6 critical genes (*CDK1*, *KIF20A*, *PBK*, *KIF2C*, *CDC20*, *UBE2C*) involved in COVID-19 Myocarditis and explored the biological processes between COVID-19 and myocarditis, confirming previous studies and providing some insights into the pathogenesis of COVID-19 Myocarditis, demonstrating that SARS-CoV-2 contributes to myocarditis through pathophysiological processes such as cell cycle and the ubiquitin-protein hydrolysis. At the same time, this study also provides relevant drugs for the clinical treatment of COVID-19 Myocarditis. There are few studies on COVID-19 Myocarditis, and if more samples are available in the future, the role of this study will be more effective in the context of the SARS-CoV-2 pandemic.

## Supporting information

S1 Text DEGs associated with COVID-19 myocarditis in GSE150392. (TXT)

S2 Text The intersection of DEGs in the GSE150392 dataset and turquoise module genes in the GSE167028. (TXT)

S3 Text GO pathways analysis results. (TXT)

S4 Text KEGG pathways analysis results. (TXT)

S5 Text COVID-19 myocarditis gene-targeted drugs. (TXT)

S1 File Transcription factor-gene regulatory network in COVID-19 myocarditis. (CYS)

## Abbreviations

adj P Val: adjusted P-Value
CMR: cardiac magnetic resonance
COVID-19: Coronavirus disease 2019
DEGs: Differentially expressed genes
DSigDB: Drug Signatures Database
EMB: endomyocardial biopsy
FC: Fold Change
GEO: Gene Expression Omnibus
GO: Gene Ontology
hiPSC-CMs: human induced pluripotent stem cell-derived cardiomyocytes
KEGG: Kyoto Encyclopedia of Genes and Genomes
PPI: protein-protein interaction
TF-gene: Transcription factor-gene
TF-miRNA: Transcription factor-microRibonucleic acid
WGCNA: weighted gene correlation network analysis
